# A phase Ib study of entinostat plus lapatinib with or without trastuzumab in patients with HER2-positive metastatic breast cancer that progressed during trastuzumab treatment

**DOI:** 10.1038/s41416-019-0473-y

**Published:** 2019-05-17

**Authors:** Bora Lim, Rashmi K. Murthy, Jangsoon Lee, Summer A. Jackson, Toshiaki Iwase, Darren W. Davis, Jie S. Willey, Jimin Wu, Yu Shen, Debu Tripathy, Ricardo Alvarez, Nuhad K. Ibrahim, Abenaa M. Brewster, Carlos H. Barcenas, Powel H. Brown, Sharon H. Giordano, Stacy L. Moulder, Daniel J. Booser, Jeffrey A. Moscow, Richard Piekarz, Vicente Valero, Naoto T. Ueno

**Affiliations:** 1 Department of Breast Medical Oncology, The University of Texas MD Anderson Cancer Center, Houston, TX USA; 2 Morgan Welch Inflammatory Breast Cancer Research Program and Clinic, The University of Texas MD Anderson Cancer Center, Houston, TX USA; 3 Methodist Hospital, Houston, TX USA; 4 ApoCell, Houston, TX USA; 5 Department of Biostatistics, The University of Texas MD Anderson Cancer Center, Houston, TX USA; 6 Southeastern Regional Medical Center, Newnan, GA USA; 7 Cancer Therapy Evaluation Program, National Cancer Institute, Rockville, MD USA

**Keywords:** Breast cancer, Breast cancer, Drug development

## Abstract

**Background:**

Human epidermal growth factor 2 (HER2) is an effective therapeutic target in breast cancer; however, resistance to anti-HER2 agents such as trastuzumab and lapatinib develops. In a preclinical model, an HDAC inhibitor epigenetically reversed the resistance of cancer cells to trastuzumab and showed synergistic efficacy with lapatinib in inhibiting growth of trastuzumab-resistant HER2-positive (HER2+) breast cancer.

**Methods:**

A phase 1b, dose escalation study was performed to assess maximum tolerated dose, safety/toxicity, clinical efficacy and explored pharmacodynamic biomarkers of response to entinostat combined with lapatinib with or without trastuzumab.

**Results:**

The combination was safe. The MTD was lapatinib, 1000 mg daily; entinostat, 12 mg every other week; trastuzumab, 8 mg/kg followed by 6 mg/kg every 3 weeks. Adverse events included diarrhoea (89%), neutropenia (31%), and thrombocytopenia (23%). Neutropenia, thrombocytopenia and hypokalaemia were noted. Pharmacodynamic assessment did not yield conclusive results. Among 35 patients with evaluable response, PR was observed in 3 patients and CR in 3 patients, 1 maintained SD for over 6 months.

**Discussion:**

This study identified the MTD of the entinostat, lapatinib, and trastuzumab combination that provided acceptable tolerability and anti-tumour activity in heavily pre-treated patients with HER2+ metastatic breast cancer, supporting a confirmatory trial.

## Background

Human epidermal growth factor receptor 2 (HER2) is overexpressed in 20–25% of all breast cancers,^1^ thus accounting for near 60,000 patients each year in the U.S.^2^ Use of trastuzumab to suppress HER2 activity in HER2-positive (HER2+) breast cancer opened up a new era of therapeutics in HER2+ breast cancers using monoclonal antibodies.^3^ However, almost all patients with HER2+ metastatic breast cancer eventually develop resistance to trastuzumab after first-line treatment; therefore, novel therapy is needed. Lapatinib co-targets both epidermal growth factor receptor (EGFR; also called HER1) and HER2, via competitive binding to the intracellular ATP binding domain of the HER2. Lapatinib (Tykerb; Novartis Pharmaceuticals Co., East Hanover, New Jersey) has shown clinical benefit in combination with capecitabine or trastuzumab, with activity noted in patients with brain metastases and multiple prior lines of treatment.^4–7^ Notably, Overall survival (OS) benefit of lapatinib combined with trastuzumab has been shown in heavily pre-treated patients with HER2+ metastatic breast cancer.^8^ Formerly, these lapatinib-based therapy combinations were implemented in the second-line setting after progression on trastuzumab plus chemotherapy.^9,10^

Histone deacetylase (HDAC) inhibitors, e.g., entinostat (SNDX-275), have been recognised as cell “switches” that can reverse the therapeutic response of cancer cells from insensitive to sensitive when combined with cancer therapeutics.^11^ Our group studied this activity of entinostat in a preclinical trastuzumab-resistant HER2+ breast cancer model, in combination with lapatinib.^12^ The drug combination had anti-tumour efficacy against HER2+ cell lines that were resistant to single-agent trastuzumab or lapatinib. In another preclinical study, entinostat enhanced the efficacy of trastuzumab in HER2-overexpressing breast cancer cells and exhibited potential to overcome trastuzumab resistance.^13^

With our preclinical study showing synergy between entinostat and anti-HER2 therapeutics as a scientific rationale, we conducted a phase Ib study investigating the novel combinations of entinostat and lapatinib plus/minus trastuzumab in patients with metastatic HER2+ breast cancer after progression during treatment with a trastuzumab combination regimen. The primary objective was to determine the maximum tolerated dose (MTD) of entinostat and lapatinib, when combined with a fixed dose of trastuzumab. The secondary objectives were determining the safety and tolerability of the combination regimen and its clinical efficacy, including the rates of complete response (CR), partial response (PR), and stable disease (SD) at 6 months from enrolment in the study. As exploratory endpoints, pharmacodynamic markers, including HER2 receptor family members EGFR, HER2 and their downstream molecule AKT, were analysed in patients’ tissue and blood samples using gene/protein detection assays.

## Methods

The trial was a single-centre, open-label, single-arm study (ClinicalTrials.gov identifier NCT01434303; NCI identifier: #8871) approved by the Investigational Drug Branch of the Cancer Therapy Evaluation Program, National Cancer Institute. The study was conducted in accordance with the Declaration of Helsinki and was approved by institutional review boards at The University of Texas MD Anderson Cancer Center. All enroled patients provided written informed consent.

### Patient eligibility

Both male and female adult patients with metastatic HER2+ breast cancer were eligible. HER2 overexpression was defined either by immunohistochemical staining intensity (3+) or fluorescence in situ hybridisation (FISH) testing (HER2/CEP17 ratio >2.0).^14^ An ECOG performance status of 0–1, normal organ function, and prior exposure to trastuzumab were required, but it was not mandated that trastuzumab to be the immediately preceding regimen. If the patient had had progression to metastatic disease within 6 months of a previous trastuzumab regimen given during treatment of local disease (e.g., adjuvant therapy), the patient was eligible. Patients had to be able to swallow and retain oral medication. Measurable disease by Response Evaluation Criteria in Solid Tumours (RECIST) v 1.1,^15^ left ventricular ejection fraction equal to or greater than 50% were required. Lactating or pregnant patients were excluded. Information about the patients’ demographic characteristics, previous lines of therapy, biomarker measurements (including oestrogen receptor and progesterone receptor) from archived tissue obtained at diagnosis or recurrence, and HER2 measurements (immunohistochemistry and FISH assay) were collected for this analysis.

### Study design

Patients were enroled in the study between [January 2012] and [November 2015]. Initially patients only received entinostat and lapatinib without trastuzumab, since the study was designed prior to the confirmed safety and efficacy of dual anti-HER2 therapy with lapatinib and trastuzumab, which were published in 2012.^8^ For the initial dose-escalation phase, patients received various doses of oral entinostat (5–15 mg every other week) and oral lapatinib (1000 or 1250 mg daily) with 28 days as one cycle and, once trastuzumab was added, the standard dose of trastuzumab: an 8 mg/m^2^ initial loading dose with subsequent doses of 6 mg/m^2^ every 3 weeks via intravenous infusion. Following the schedule of oral regimens, 28 days were considered as one cycle. A 3 + 3 design, in which each new dose level was tested in 3 consecutive patients, was used to determine the MTD (dose level summarised in Table 2). After the MTD was determined, an additional 10 evaluable patients were enroled as an expansion cohort to confirm the treatment safety and tolerability. No diarrhoeal prophylaxis was introduced.

### Dose modification and toxicity assessment

Adverse events (AEs) and laboratory results were graded according to the National Cancer Institute Common Terminology Criteria for Adverse Events v 4.03.^16^ Dose-limiting toxicity (DLT) was defined as one of the following AEs with an attribution of possibly, probably, or definitely related to the study agents and occurring within 28 days after the first dose: grade 4 neutropenia lasting >7 days or any febrile neutropenia; grade 4 thrombocytopenia; non-haematologic toxicity ≥grade 3; or >14 days of treatment delay due to any therapy-related toxicity of any grade. Nausea/vomiting, diarrhoea, and electrolyte imbalances were considered DLT if they persisted for 48 h despite adequate supportive care. Toxicity was evaluated on days 15 and 28 for first 2 cycles, and at the end of each cycle thereafter.

### Efficacy evaluation

Tumour assessments were conducted based on RECIST v1.1.^15^ Clinical efficacy assessment measured the patient’s best response: complete response (CR), partial response (PR), stable disease (SD), or progressive disease (PD) after the first 2 cycles and every 2 cycles subsequently unless there was a clear progression on skin in patients with inflammatory breast cancer (IBC). The clinical benefit rate was defined as the percentage of patients combined who had SD lasting at least 6 months, PR, or CR. For survival analysis, OS and PFS were measured from the day the patients started trial drugs to the times the patients died or had disease progression, respectively. OS was assessed based on death reports and last available follow-up in the clinic as of April 6, 2017 when the final analysis was performed. For patients who had obvious clinical progression prior to the first scan, the date of clinical progression was annotated as the date of progression.

### Pharmacodynamic markers

For exploratory biomarker analysis, archived tumour samples obtained from biopsies and prospectively collected blood samples were analysed using at Apocell, Inc. (Houston, TX). Tissue samples were analysed for protein expression of EGFR, HER2, and AKT and their phosphorylated forms, and for gene levels of EGFR and HER2. The expression of each gene was measured by FISH. Circulating tumour cells (CTCs) from peripheral blood were collected at baseline and after cycle 1. The Wilcoxon signed-rank test was used to examine the change in target molecule expression measures from baseline to after cycle 1. While blood-based markers including CTC were collected before and after the therapeutic intervention, tissues were collected retrospectively, thus mostly baseline biopsy of surgical samples were utilised for PD tissue biomarker analysis.

### Statistical analysis

Data were summarised using standard descriptive statistics such as mean, standard deviation, median, and range for continuous variables and frequency and proportion for categorical variables. Association between categorical variables was examined by the chi-square test or Fisher exact test when appropriate. The Wilcoxon rank-sum test was used to examine differences in continuous variables between patient characteristic groups. OS time and PFS time were estimated using the Kaplan-Meier method, and the comparison between or among patient characteristic groups was evaluated by the log-rank test. Univariate and multivariate Cox regression models were applied to assess the effect of covariates of interest on OS and PFS. Computations were carried out in SAS 9.3 (SAS Institute Inc., Cary, NC, USA) and R 3.2.4.

## Results

### Patient characteristics

Among 37 patients who signed the consent form and enroled in the study, 1 patient was found prior to starting therapy to have negative HER2 status on institutional pathology review, and another patient was admitted to the hospital for a non-trial-related severe illness and did not pursue further treatment after the first several doses. The remaining 35 patients were evaluable for study endpoints. Demographic characteristics are summarised in Table 1. Only 1 patient was male. Twenty-four were white, 6 were Hispanic, and 5 were black. The median age was 52 years (range: 26–72).

**Table 1:** Demographic and clinical characteristics for the full patient cohort and each treatment group: categorical variables

Covariate	Levels	Total (*N* = 35)	Treatment Groups	
			Entinostat + Lapatinib (*N* = 14)	Entinostat + Lapatinib + Trastuzumab (*N* = 21)
Sex	Female	34 (97.1%)	13 (38.2%)	21 (61.8%)
	Male	1 (2.9%)	1 (100%)	0 (0%)
Race/Ethnicity	Black	5 (14.3%)	1 (20%)	4 (80%)
	Hispanic	6 (17.1%)	4 (66.7%)	2 (33.3%)
	White	24 (68.6%)	9 (37.5%)	15 (62.5%)
ER	Positive	17 (48.6%)	6 (35.3%)	11 (64.7%)
	Negative	18 (51.4%)	8 (44.4%)	10 (55.6%)
	Negative	25 (73.5%)	10 (40%)	15 (60%)
PgR	Positive	9 (26.5%)	4 (44.4%)	5 (55.6%)
	Unknown	1 (2.8%)		
HER2 IHC	3+	5 (100%)	2 (40%)	3 (60%)
	Unknown	30 (85.7%)		
Histopathology	Inflammatory breast cancer	13 (37.1%)	6 (46.2%)	7 (53.8%)
	Invasive ductal carcinoma	22 (62.9%)	8 (36.4%)	14 (63.6%)
Previous anti-HER2 therapy	Trastuzumab*	35 (100%)	14 (100%)	21 (100%)
	Pertuzumab	15 (42.9%)	0 (0%)	15 (100%)
	T-DM1	19 (54.3%)	2 (10.5%)	17 (89.5%)
	Lapatinib	14 (40%)	7 (50%)	7 (50%)
Metastatic Site	Bone	4 (11.4%)	0 (0%)	4 (11.4%)
	Chest wall	5 (14.2%)	1 (2.8%)	4 (11.4%)
	Liver	6 (17.1%)	0 (0%)	6 (17.1%)
	Lung	7 (20%)	0 (0%)	7 (20%)
	Lymph nodes	5 (14.2%)	0 (0%)	5 (14.2%)
	Pancreas	1 (2.8%)	0 (0%)	1 (2.8%)

*ER* oestrogen receptor, *PgR* progesterone receptor, *IHC* immunohistochemistry, *T-DM1* ado-trastuzumab emtansine. *All but 1 patient received prior trastuzumab, pertuzumab, T-DM1, and lapatinib in the metastatic setting. However, 1 patient received trastuzumab in the adjuvant setting; when the patient’s localised disease progressed to metastatic within 6 months, the patient enroled in the trial

The median number of previous lines of therapy for metastatic disease was 3 (range: 0–15). The median best progression-free survival (PFS) on a prior trastuzumab-containing regimen for all patients was 4 months (range: 0–33). The mean duration of prior trastuzumab exposure was 14.5 months, and the maximum duration of response to trastuzumab had been 77 months prior to study.

### Dose escalation and MTD

The first 14 patients received entinostat and lapatinib in combination. Eleven patients received entinostat, lapatinib, and trastuzumab in combination until the MTD was reached. Once the MTD was defined, 10 additional patients were enroled and treated. The median number of cycles of treatment was 3 (counting 28 days as 1 cycle). The median follow-up time was 2.53 years (95% CI: 1.77, 4.30).

No patients in the entinostat/lapatinib group experienced DLT. Among patients who received all three agents in combination, 2 of 5 patients experienced DLT at 15 mg of entinostat, 1000 mg of lapatinib, and standard-dose trastuzumab; 1 patient had grade 3 thrombocytopenia, and 1 patient had grade 3 diarrhoea that required dose reduction. Thus, the MTD was designated as one dose level down (Table 2, cohort 6): 12 mg of entinostat, the same 1000-mg dose of lapatinib, and standard-dose trastuzumab, and the cohort was expanded to 6. One of 6 patients treated at the MTD had grade 4 hypokalaemia that required hospitalisation. Among the 10 out of 11 evaluable patients who were enroled in the expansion cohort (excluding 1 patient who was not evaluable because the patient withdrew the consent before reaching 28 days), 1 patient experienced grade 3 diarrhoea consistent with DLT during the first 28 days.

**Table 2:** Dose-limiting toxicities in dose-escalation cohorts

	Cohort	Entinostat dose every other week (mg)	Lapatinib once daily (mg)	Trastuzumab every 3 weeks (mg/kg)	Patients, *n*	Patients with DLT, *n*	DLT(s)
Dose level	1	5	1250	NA	3	0	
	2	8	1250	NA	3	0	
	3	10	1250	NA	3	0	
	4	12	1250	NA	3	0	*
	5	15	1250	NA	2	0	
	6	12	1000	8->6	6	1	Hypokalaemia
	7	15	1000	8->6	5	2	Thrombocytopenia, diarrhoea
Expansion	6	12	1000	8->6	10	1	Diarrhoea

*DLT* dose-limiting toxicity, *NA* not applicable. *One patient from cohort 4 withdrew after confirmation of negative HER2 status but did not have DLT, and the next patient was accrued to the next dose level cohort

### Safety and tolerability of combination

All patients experienced at least grade 1 AEs during the study and experienced a total of 244 treatment-related AEs (Table 3; Supplemental Table S1). Of the 21 patients who received entinostat, lapatinib, and trastuzumab in combination, grade 3 neutropenia, leukopenia, thrombocytopenia were observed throughout the treatment. One had grade 4 thrombocytopenia. Three patients developed infections. Several electrolyte abnormalities were observed. Of the 14 patients who received entinostat and lapatinib without trastuzumab, grade 3 neutropenia and thrombocytopenia were observed.

**Table 3:** Adverse events for the full patient cohort and each treatment group

Adverse Event	All Patients (*N* = 35)		Entinostat + Lapatinib + Trastuzumab (Patient: *N* = 21)		Entinostat + Lapatinib (Patient: *N* = 14)	
	All Grades *N* (%)	Grade 3-4 *N* (%)	All Grades *N* (%)	Grade 3-4 *N* (%)	All Grades *N* (%)	Grade 3-4 *N* (%)
Any Adverse Event	35 (100)	28 (80)	21 (100)	18 (85.7)	14 (100)	10 (71.4)
Abdominal pain	2 (5.7)	2 (5.7)	1 (4.8)	1 (4.8)	1 (7.1)	1 (7.1)
Alkaline phosphatase increased	1 (2.9)	1 (2.9)	0 (0)	0 (0)	1 (7.1)	1 (7.1)
Anaemia	5 (14.3)	5 (14.3)	5 (23.8)	5 (23.8)	0 (0)	0 (0)
Arthralgia	1 (2.9)	1 (2.9)	0 (0)	0 (0)	1 (7.1)	1 (7.1)
Blood bilirubin increased	1 (2.9)	1 (2.9)	1 (4.8)	1 (4.8)	0 (0)	0 (0)
Dehydration	1 (2.9)	1 (2.9)	0 (0)	0 (0)	1 (7.1)	1 (7.1)
Diarrhoea	31 (88.6)	4 (11.4)	18 (85.7)	4 (19)	13 (92.9)	0 (0)
Dyspnoea	3 (8.6)	3 (8.6)	0 (0)	0 (0)	3 (21.4)	3 (21.4)
Fatigue	8 (22.6)	8 (22.6)	3 (14.3)	3 (14.3)	5 (35.7)	5 (35.7)
Hyperglycaemia	1 (2.9)	1 (2.9)	0 (0)	0 (0)	1 (7.1)	1 (7.1)
Hypocalcaemia	1 (2.9)	1 (2.9)	1 (4.8)	1 (4.8)	0 (0)	0 (0)
Hypokalaemia	3 (8.6)	3 (8.6)	3 (14.3)	3 (14.3)	0 (0)	0 (0)
Hyponatraemia	2 (5.7)	2 (5.7)	1 (4.8)	1 (4.8)	1 (7.1)	1 (7.1)
Lung infection	1 (2.9)	1 (2.9)	1 (4.8)	1 (4.8)	0 (0)	0 (0)
Myalgia	1 (2.9)	1 (2.9)	1 (4.8)	1 (4.8)	0 (0)	0 (0)
Neoplasms benign, malignant and unspecified (incl cysts and polyps) - (Other), specify^a^	2 (5.7)	2 (5.7)	1 (4.8)	1 (4.8)	1 (7.1)	1 (7.1)
Neutrophil count decreased	11 (31.4)	11 (31.4)	8 (38.1)	8 (38.1)	3 (21.4)	3 (21.4)
Pain of skin	1 (2.9)	1 (2.9)	0 (0)	0 (0)	1 (7.1)	1 (7.1)
Platelet count decreased	8 (22.6)	8 (22.6)	6 (28.6)	6 (28.6)	2 (14.3)	2 (14.3)
Rash maculo-papular	1 (2.9)	1 (2.9)	0 (0)	0 (0)	1 (7.1)	1 (7.1)
Sepsis	1 (2.9)	1 (2.9)	1 (4.8)	1 (4.8)	0 (0)	0 (0)
Urinary tract infection	1 (2.9)	1 (2.9)	1 (4.8)	1 (4.8)	0 (0)	0 (0)
White blood cell decreased	3 (8.6)	3 (8.6)	3 (14.3)	3 (14.3)	0 (0)	0 (0)
Any Treatment-Related Adverse Event	34 (97.1)	28 (80)	20 (95.2)	18 (85.7)	14 (100)	10 (71.4)
Abdominal pain	2 (5.7)	2 (5.7)	1 (4.8)	1 (4.8)	1 (7.1)	1 (7.1)
Alkaline phosphatase increased	1 (2.9)	1 (2.9)	0 (0)	0 (0)	1 (7.1)	1 (7.1)
Anaemia	5 (14.3)	5 (14.3)	5 (23.8)	5 (23.8)	0 (0)	0 (0)
Arthralgia	1 (2.9)	1 (2.9)	0 (0)	0 (0)	1 (7.1)	1 (7.1)
Blood bilirubin increased	1 (2.9)	1 (2.9)	1 (4.8)	0 (0)	0 (0)	0 (0)
Dehydration	1 (2.9)	1 (2.9)	0 (0)	0 (0)	1 (7.1)	1 (7.1)
Diarrhoea	31 (88.6)	4 (11.4)	18 (85.7)	4 (85.7)	13 (92.9)	0 (0)
Dyspnoea	3 (8.6)	3 (8.6)	0 (0)	0 (0)	3 (21.4)	3 (21.4)
Fatigue	8 (22.6)	8 (22.6)	3 (14.3)	3 (14.3)	5 (35.7)	5 (35.7)
Hyperglycaemia	1 (2.9)	1 (2.9)	0 (0)	0 (0)	1 (7.1)	1 (7.1)
Hypocalcaemia	1 (2.9)	1 (2.9)	1 (4.8)	1 (4.8)	0 (0)	0 (0)
Hypokalaemia	3 (8.6)	3 (8.6)	3 (14.3)	3 (14.3)	0 (0)	0 (0)
Hyponatraemia	2 (5.7)	2 (5.7)	1 (4.8)	1 (4.8)	1 (7.1)	1 (7.1)
Lung infection	1 (2.9)	1 (2.9)	1 (4.8)	1 (4.8)	0 (0)	0 (0)
Myalgia	1 (2.9)	1 (2.9)	1 (4.8)	1 (4.8)	0 (0)	0 (0)
Neutrophil count decreased	11 (31.4)	11 (31.4)	8 (38.1)	8 (38.1)	3 (21.4)	3 (21.4)
Pain of skin	1 (2.9)	1 (2.9)	0 (0)	0 (0)	1 (7.1)	1 (7.1)
Platelet count decreased	8 (22.6)	8 (22.6)	6 (28.6)	6 (28.6)	2 (14.3)	2 (14.3)
Rash maculo-papular	1 (2.9)	1 (2.9)	0 (0)	0 (0)	1 (7.1)	1 (7.1)
Sepsis	1 (2.9)	1 (2.9)	1 (4.8)	1 (4.8)	0 (0)	0 (0)
Urinary tract infection	1 (2.9)	1 (2.9)	1 (4.8)	1 (4.8)	0 (0)	0 (0)
White blood cell decreased	3 (8.6)	3 (8.6)	3 (14.3)	3 (14.3)	0 (0)	0 (0)

^a^Neoplasms benign, malignant and unspecified: one in each arm is the only grade 5 adverse event that is unrelated with treatment

Diarrhoea was the most frequently reported AE, seen in 31 patients (89%). Not surprisingly given that the starting dose of lapatinib was higher in the first 14 patients who received entinostat and lapatinib alone without trastuzumab, the frequency of diarrhoea was higher in that group: 13 of 14 patients (92.9%) compared to 18 of 21 (85.7%) in the triple combination group (Table 3). Among the patients with reported diarrhoea, 27 patients had grade 1–2 diarrhoea, while 4 patients had grade 3. One patient withdrew consent after 4 months on the study due to intolerance from diarrhoea.

### Efficacy

**Fig. 1:**
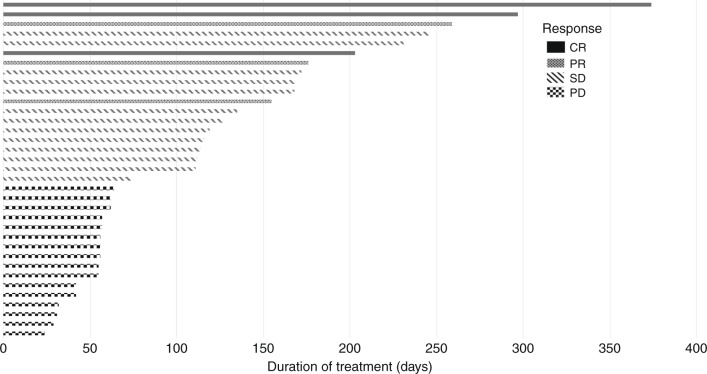
Swimmer plot showing clinical response for each patient. Each bar represents an individual patient. Different clinical responses are indicated by different patterns, as shown in the legend on the right

The overall clinical benefit rate was 20%: 3 patients with CR, 3 patients with PR, and 1 patient who maintained SD for over 6 months (Table 4). A total of 17 patients (48.6%) had PD as the best response. The clinical benefit rate in IBC patients was not inferior to that in non-IBC patients (23.1 vs 18.2%, respectively; *P* = 1). There was no significant difference in clinical benefit rate between patients who had received previous lapatinib use *vs* not, pertuzumab *vs* not, and T-DM1 *vs* not. The best response for each patient is summarised in a swimmer plot in Fig. 1.

**Table 4:** Patients’ best response during the treatment period for the full patient cohort and each treatment group

Best Response	Total (*N* = 35)	Treatment Groups		*P*-value
		Entinostat + Lapatinib (*N* = 14)	Entinostat + Lapatinib + Trastuzumab (*N* = 21)	
PD	17 (48.6%)	9 (64.3%)	8 (38.1%)	0.1756
No PD	18 (51.4%)	5 (35.7%)	13 (61.9%)	
No PD				
CR	3 (8.6%)	1 (7.1%)	2 (9.5%)	
PR	3 (8.6%)	0 (0%)	3 (14.3%)	
SD	12 (34.3%)	4 (28.6%)	8 (38.1%)	
CB*	7 (20%)	1 (7.1%)	6 (28.6%)	0.2027
No CB	28 (80%)	13 (92.9%)	15 (71.4%)	
CB*				
CR	3 (8.6%)	1 (7.1%)	2 (9.5%)	
PR	3 (8.6%)	0 (0%)	3 (14.3%)	
SD ≥ 6 months	1 (2.9%)	0 (0%)	1 (4.8%)	

Best Response	Total (*N* = 35)	Treatment Groups		*P*-value**
		Entinostat + Lapatinib (*N* = 14)	Entinostat+ Lapatinib+ Trastuzumab (*N* = 21)	
CR	3 (8.6%)	1 (7.1%)	2 (9.5%)	
PR	3 (8.6%)	0 (0%)	3 (14.3%)	
SD	12 (34.3%)	4 (28.6%)	8 (38.1%)	
≥6 months	1 (2.9%)	0 (0%)	1 (4.8%)	
PD	17 (48.6%)	9 (64.3%)	8 (38.1%)	0.1756
CB*	7 (20%)	1 (7.1%)	6 (28.6%)	0.2027

*PD* progressive disease, *CR* complete response, *PR* partial response, *SD* stable disease, *CB* clinical benefit

*CB was defined as CR, PR, or SD ≥ 6 months

**Comparison of presence vs absence of the indicated response in the full patient cohort by student *T*- test

**Fig. 2:**
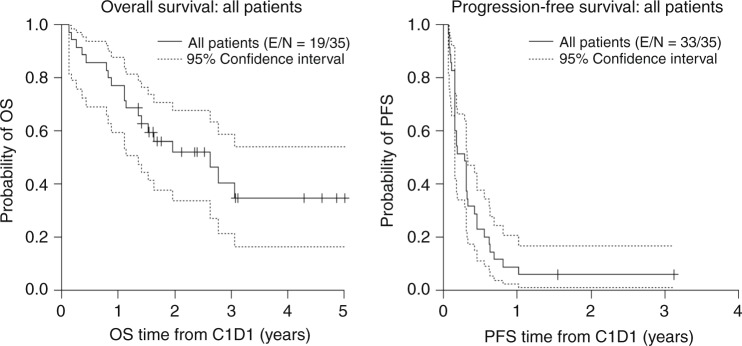
Overall survival and progression-free survival of all patients from cycle 1, day 1 (C1D1) of study treatment. E/N, Number of patients with the event (death for OS and disease progression for PFS) / Total number of evaluable patients. Among the 35 patients, 19 (54.2%) died. The median OS time was 2.63 years (95% CI: 1.36-NA), and the median follow-up time was 2.53 years (95% CI: 1.77, 4.30)

PFS and OS curves for the full cohort are shown in Fig. 2. The median PFS time was 3.48 months (95% CI: 1.81, 3.78). Analysis by oestrogen receptor and progesterone receptor positivity, IBC *vs* non-IBC, and prior therapies did not show association of these factors with PFS.

As of final data analysis (April 6, 2017), over half of the patients had died (54.2%, 19/35). Two patients died within 30 days of study completion. Both patients were confirmed to have PD at day 42 of the study enrolment prior to the first staging scan by other exams. The median OS was 2.63 years (95% CI: 1.36, NA). Age was found to be significantly associated with OS (HR = 1.07; 95% CI: 1.02, 1.12; *P* = 0.0071). There was a favourable but not statistically significant trend for PFS in patients who had previously received pertuzumab. Other variables (race, receptor status, IBC vs non-IBC, and prior therapies) were not associated with OS. OS rates at 1, 2, and 5 years were 77%, 52%, and 35%, respectively.

### Pharmacodynamics

The median FISH HER2/CEP17 ratios were 7.84 in the entinostat/lapatinib treatment group *vs* 5.5 in the entinostat/lapatinib/trastuzumab group; however, the difference was not statistically significant (Supplementary Table S2). In 20 of 35 patients (57.1%), more than 10% of cells were positive for oestrogen receptor and/or progesterone receptor expression.

A total of 23 baseline tissue samples were analysed for HER2, EGFR, AKT, and the phosphorylated forms of these proteins, as well as the HER2 and EGFR genes by FISH. No statistical significance was found for these markers with regard to clinical efficacy.

A total of 52 CTC samples were obtained and analysed for correlative biomarkers in 20 patients from the entinostat/lapatinib/trastuzumab treatment group, among whom 17 had paired samples collected at baseline and after the first cycle of treatment. For these 17 patients, the median time from cycle 1, day 1, to the date of the second CTC measurement was 28 days (Supplemental Table S3). Neither baseline CTC counts or changes in CTCs and CTC-based biomarkers showed significant correlation with clinical efficacy.

## Discussion

We report results of a phase Ib trial evaluating the combination of entinostat, lapatinib, and trastuzumab at a single institution. The MTD was defined, and the treatment was safe and well tolerated, with mainly grade 1 and 2 toxicities. There was also encouraging early clinical benefit noted in this heavily pre-treated patient population.

The side effect profile of HDAC inhibitors includes pancytopenia, and the potential for enhancing off-target effects (i.e., cardiac toxicity or diarrhoea) of lapatinib and/or trastuzumab was anticipated. In our study, diarrhoea was the most common side effect. Major DLTs included grade 3 and 4 haematologic toxicities and grade 3 diarrhoea. Despite the known high frequency of high-grade diarrhoea induced by lapatinib, the combination with entinostat did not result in high-grade diarrhoea; the rate of grade 3 or above diarrhoea was lower compared to previous lapatinib-related studies,^17–19^ which was positive aspect to this combination. However, the rate of high grade diarrhoea remains to be significantly high at 19% grade 3 or above, that further needs to be confirmed in larger size trials. Whether the epigenetic modulation of entinostat contributes to this lower rate of diarrhoea is unclear and should be studied further. We did not observe any cases of cardiac toxicity during the trial period. One case of grade 4 hypokalaemia was observed without treatment-related diarrhoea, and the correlation between the therapy and the occurrence of hypokalaemia was unclear.

HDAC inhibitors have been recognised to have a role in reversing resistance to anti-HER2 therapies.^13^ Indeed, entinostat has shown the ability to reverse resistance to endocrine therapy in hormone receptor-positive breast cancer,^20^ and currently being studied in hormone receptor-positive metastatic breast cancer in combination with an aromatase inhibitor in a phase III trial (NCT02115282). In our preclinical models, addition of HDAC inhibitor induced the apoptotic process in reversing the resistance to trastuzumab,^12^ and this is the first study to report entinostat and anti-HER2 therapy combination in the clinical setting, to our knowledge.

The encouraging clinical benefit rate of 20% in a heavily pre-treated population is of interest for further study. Patients with multiple previous lines of treatment still showed clinical benefit. There was no significant difference based on previous use of T-DM1. On the other hand, previous pertuzumab use had a trend of association with longer OS, although the difference was not statistically significant. We can hypothesise that when HER2+ breast cancer has already been treated by co-inhibition of heterodimerization partner HER3 by the use of pertuzumab and developed resistance, then another partner of the HER2 receptor, EGFR, may be overexpressed as a compensatory mechanism to the therapeutics, in which case, an anti-EGFR inhibitor maybe more effective compare to lapatinib. The efficacy was also shown in patients who had previous lapatinib containing regimen, which suggests a role of entinostat in reversal of resistance. Although lapatinib has shown activity against HER2 positive breast cancer that has metastasised to brain, we designed to exclude patients given the concern over rapid progression of patients with brain metastasis and the heterogeneous patient population that could potentially affect our clinical outcome analysis. To test this, a separate clinical trial dedicated to study an efficacy for patients with brain metastasis using our combination can be considered.

We analysed CTCs and tissue-based biomarkers to correlate changes in expression among key EGFR/HER2-PI3K-AKT pathway molecules with clinical efficacy, however we could not show meaningful association with clinical outcome. There were several limitations with regard to the CTC results. First, the collection of CTCs was mandated only during the triple-therapy phase of the trial; therefore, only 17 had CTCs collected from both baseline and after the first cycle of treatment, and 5 among those did not have detectable CTCs. Another limitation was the method of CTC detection. In 2010, EpCAM-negative (mesenchymal) CTCs were not included among the detected cells from the blood, which may have lowered the sensitivity of detection. Moreover, while the number of CTCs in patients with metastatic breast cancer was shown to correlate with long term survival,^21,22^ changes made by treatment, including our proposed entinostat-containing combination therapy has never been investigated before. In addition, the BEACON trial demonstrated significant correlations in mechanistic biomarkers in CTCs and overall survival.^23^ Circulating tumour DNA has shown its value as a real-time predictor of resistance to ongoing therapy in metastatic breast cancer before,^24^ however our study mainly tested CTC instead of ctDNA for two reasons. First, at the time of our original study development, ctDNA assay was not readily available due to sensitivity issue. Second and more importantly, we used CTC to detect protein changes, which is limited when ctDNA is used as liquid biopsy tools given detection of mutation-based data. In terms of the changes in the number of CTC, we hypothesised that effective therapy in metastatic breast cancer can induce the reduction of peripheral CTCs, thus can be utilised as a biomarker of response as itself while it enables the functional changes of circulating markers based on protein changes. This hypothesis needs further investigation in future studies with larger number of samples. Lastly, given limited available cells for testing, we had to omit two important biomarkers of HER2 resistance, HER3 and IGF-1R as critical biomarkers on CTC, and this is another limitation of our biomarker analysis. We plan to study these markers, as well as other biomarkers that have shown to confer resistance, e.g., Bim in our future studies. Taken together, although the analysis did not reveal any correlations with many limitations as we summarised here, we did demonstrate the feasibility of using CTC-based testing as a complement to tissue-based testing in future trials.

The primary objective of assessing the safety and MTD of the novel drug combination was fulfilled. Completing this assessment of clinical efficacy requires a larger phase II study of entinostat in combination with a HER2-targeted agent as a therapeutic option for trastuzumab-resistant HER2+ metastatic breast cancer, and while there were limitations in clinical efficacy assessment and biomarker, we believe our phase Ib data supports the rationale to conduct such trial. To strengthen the data on potential relevant biomarkers, we plan to analyse the mechanism of action of entinostat and serum-based biomarker expression changes in future trials.

## Supplementary information

Supplementary data

## Data Availability

All data generated or analysed during this study are included in this published article [and its supplementary information files].
