# Supplementary material for: A phase Ib study of entinostat plus lapatinib with or without trastuzumab in patients with HER2-positive metastatic breast cancer that progressed during trastuzumab treatment
Source: Br J Cancer. 2019 May 17;120(12):1105–12. doi: 10.1038/s41416-019-0473-y (PMC6738035; doi:10.1038/s41416-019-0473-y)
Supplement: Supplementary file 1 — Supplementary data [file 41416_2019_473_MOESM1_ESM.docx]

**Supplementary Tables and Figures**

| Supplementary Table S1. Adverse events by grade and attribution of their relationships to the study treatments | | | | |
| --- | --- | --- | --- | --- |
| Grade | Relationship to Treatment Regimen | Number of Events | | |
|  |  | Total  (N=35) | Treatment Groups | |
|  |  |  | Entinostat+Lapatinib | Entinostat+Lapatinib+Trastuzumab |
|  |  |  | (N=14) | (N=21) |
| 5 | Definite | 0 | 0 | 0 |
|  | Probable | 0 | 0 | 0 |
|  | Possible | 0 | 0 | 0 |
|  | Unlikely | 1 | 0 | 1 |
|  | Unrelated | 1 | 1 | 0 |
|  | **GRADE 5 TOTALS** | **2** | **1** | **1** |
|  |  |  |  |  |
| 4 | Definite | 0 | 0 | 0 |
|  | Probable | 6 | 3 | 3 |
|  | Possible | 5 | 1 | 4 |
|  | Unlikely | 0 | 0 | 0 |
|  | Unrelated | 0 | 0 | 0 |
|  | **GRADE 4 TOTALS** | **11** | **4** | **7** |
|  |  |  |  |  |
| 3 | Definite | 3 | 1 | 2 |
|  | Probable | 64 | 26 | 38 |
|  | Possible | 20 | 2 | 18 |
|  | Unlikely | 1 | 0 | 1 |
|  | Unrelated | 0 | 0 | 0 |
|  | **GRADE 3 TOTALS** | **88** | **29** | **59** |
|  |  |  |  |  |
| 2 | Definite | 2 | 0 | 2 |
|  | Probable | 30 | 3 | 27 |
|  | Possible | 10 | 10 | 0 |
|  | Unlikely | 0 | 0 | 0 |
|  | Unrelated | 0 | 0 | 0 |
|  | **GRADE 2 TOTALS** | **42** | **13** | **29** |
|  |  |  |  |  |
| 1 | Definite | 5 | 1 | 4 |
|  | Probable | 58 | 1 | 57 |
|  | Possible | 38 | 38 | 0 |
|  | Unlikely | 0 | 0 | 0 |
|  | Unrelated | 0 | 0 | 0 |
|  | **GRADE 1 TOTALS** | **101** | **40** | **61** |
|  |  |  |  |  |
|  | **Total** | **244** | **87** | **157** |

| Supplementary Table S2. Demographic and clinical characteristics for the full patient cohort and each treatment group: continuous variables | | | | | | | |
| --- | --- | --- | --- | --- | --- | --- | --- |
| Covariate | Treatment Groups | N | Median | Range | Mean | SD | *P*-value |
| Age at Consent, years | All Patients | 35 | 52 | (26, 72) | 51.77 | 12.72 |  |
|  | Entinostat+Lapatinib | 14 | 51.5 | (26, 67) | 49.64 | 13.07 | 0.5370 |
|  | Entinostat+Lapatinib+Trastuzumab | 21 | 55 | (30, 72) | 53.19 | 12.59 |  |
| FISH HER2/CEP Ratio | All Patients | 31 | 5.5 | (2, 15.1) | 6.75 | 3.57 |  |
|  | Entinostat+Lapatinib | 12 | 7.84 | (2.64, 11.76) | 7.60 | 3.31 | 0.2821 |
|  | Entinostat+Lapatinib+Trastuzumab | 19 | 5.5 | (2, 15.1) | 6.22 | 3.71 |  |
| # of Cycles of Study Treatment Completed | All Patients | 35 | 3 | (1, 13) | 3.83 | 2.93 |  |
|  | Entinostat+Lapatinib | 14 | 2 | (1, 13) | 3.50 | 3.44 | 0.2577 |
|  | Entinostat+Lapatinib+Trastuzumab | 21 | 4 | (1, 10) | 4.05 | 2.60 |  |

| Supplementary Table S3. Number of identified CTCs processed for the measurement of each marker | | | |
| --- | --- | --- | --- |
|  | N | | |
|  | Baseline | After Cycle | Both Time Points |
| Panel 1 |  |  |  |
| P1_Total HER2 MFI* | 7 | 10 | 5 |
| P1_Phospho HER2 MFI | 7 | 10 | 5 |
| P1_Ratio of Phospho HER2 Over Total HER2 | 7 | 10 | 5 |
| Panel 2 |  |  |  |
| P2_Total EGFR MFI | 8 | 5 | 4 |
| P2_Phospho EGFR MFI | 8 | 5 | 4 |
| P2_Ratio of Phospho EGFR Over Total EGFR | 8 | 5 | 4 |
| Panel 3 |  |  |  |
| P3_Total AKT MFI | 8 | 8 | 4 |
| P3_Phospho AKT MFI | 8 | 8 | 4 |
| P3_Ratio of Phospho AKT Over Total AKT | 8 | 8 | 4 |
|  |  |  |  |
| HER2 FISH Phenotype on Cells | 16 | 11 | 9 |
| EGFR FISH Phenotype on Cells | 16 | 10 | 9 |
| *Each panel indicates the number of CTCs with measurable level of proteins of interest using micro flow imaging intensity (MFI) assay. The right column indicates that there were fewer cells used for measurement of each protein. The actual measurements of each protein of interest are summarized in Supplementary Table S7.2. | | | |

Supplementary Figure S1. Overall survival and progression-free survival analysis by treatment group
